# Glycated hemoglobin predicts coronary artery disease in non-diabetic adults

**DOI:** 10.1186/s12872-019-01302-5

**Published:** 2019-12-21

**Authors:** Mohammed Ewid, Hossam Sherif, Syed Muhammad Baqui Billah, Nazmus Saquib, Wael AlEnazy, Omer Ragab, Saed Enabi, Tawfik Rajab, Zaki Awad, Rami Abazid

**Affiliations:** 1 College of Medicine, Sulaiman Al Rajhi Colleges, P.O. Box 777, Al Bukayriah, Qassim 51941, Saudi Arabia; 2 Internal Medicine Department, Faculty of Medicine, Cairo University, Cairo, 11562 Egypt; 3 Critical Care Medicine Department, Faculty of Medicine, Cairo University, Critical Care, Kasr A. Ainy St, Cairo, 11562 Egypt; 4 Prince Sultan Cardiac Center, Ministry of Health, Al Qassim, Qassim 52366, Buraydah, 7430 An Naziyah Saudi Arabia; 5 Department of Nuclear Medicine, London Health Sciences Center, 800 Commissioners Road East, PO Box 5010, London, ON N6A 5W9 Canada

**Keywords:** HbA1c, Coronary artery disease, CAD risk factors

## Abstract

**Background:**

Coronary artery disease (CAD) is a major cause of morbidity and mortality worldwide. Due to increased CAD risk factors in Saudi Arabia, research on more feasible and predictive biomarkers is needed. We aimed to evaluate glycated hemoglobin (HbA1c) as a predictor of CAD in low-risk profile non-diabetic patients living in the Al Qassim region of Saudi Arabia.

**Methods:**

Thirty-eight patients with no history of CAD were enrolled in this cross-sectional study. They provided demographic data, and their HbA1c estimation followed the National Glycohemoglobin Standardization Program parameters. All patients underwent coronary computed tomography angiography (CCTA) for evaluation of chest pain. The extent of coronary artery stenosis (CAS) was quantified as percentage for each patient based on plaques detected in CCTA.

**Results:**

Mean blood pressure of the patients was (91.2 ± 11.9 mmHg), BMI (28.3 ± 5.8 kg/m^2^), serum cholesterol level (174 ± 33.1 mg/dl), and HbA1c levels (mean 5.7 ± 0.45, median 5.7 and range 4.7–6.4%). Eighteen patients showed no CAS (47.4%), 12 showed minimal stenosis (31.6%), 3 showed mild stenosis (7.9%), 3 showed moderate stenosis (7.9%) and 2 showed severe stenosis (5.3%). A moderate correlation was detected between HbA1c and CAS percentages (*r* = 0.47, *p* < 0.05) as well as between HbA1c and the number of affected coronary vessels (*r* = 0.53, *p* < 0.001).

**Conclusion:**

Glycated hemoglobin can be used as a predictive biomarker for CAD in non-diabetic low-risk patients.

## Background

Coronary artery disease (CAD) is a major health problem in Saudi Arabia, as is the case internationally; an estimated increase of 6.3 million CAD-related deaths is expected worldwide between 2008 and 2030 [1]. This increasing death trend is also true for the middle- and high-income countries belonging to the Gulf Cooperation Council, including Saudi Arabia [2].

The prevalence of major cardiovascular disease (CVD) risk factors, such as diabetes, obesity, hypertension, and physical inactivity, has increased in Saudi Arabia recently. Over the last two decades, obesity has increased from 35.6 to 65.5%, hypertension from 26.1 to 40%, and type 2 diabetes from 10.6 to 32.1% [3, 4]. It is speculated that rapid economic transition, urbanization, and increased prevalence of CVD risk factors in the population are some of the reasons for the increase of CAD [5].

An effective way to curb the adverse outcomes (i.e., complications, mortality) of a chronic disease like CAD is to diagnose it early in its development and preferably by non-invasive tools. At present, there are a number of screening tools available for CAD, namely high-sensitivity C-reactive protein, lipoprotein-associated phospholipase A2, ankle-brachial index, and multiple imaging studies like coronary computed tomography angiography (CCTA) with coronary artery calcium scoring [6].

Each of the screening tools has its advantages and disadvantages. Unfortunately, there is no settled consensus among clinicians on which tool is more likely to accurately predict fatal and non-fatal CAD-related outcomes [7]. Therefore, there is room for assessment of additional biomarkers that can detect early metabolic changes related to atherosclerosis and CAD.

Glycated hemoglobin (HbA1c), a well-known biomarker that reflects long-term glycemic control, has been an established diagnostic test for diabetic patients since 2010 [8]. Its value for the prediction of microvascular and macrovascular complications among diabetic patients is well established [8]. However, its potential as a screener of CAD among non-diabetic patients has shown mixed results in the literature.

This controversy in the literature regarding HbA1c is still ongoing. There are recent studies that did not find a positive correlation between HbA1c and cardiovascular-related outcomes (for example, death, nonfatal myocardial infarction, stroke, or hospitalization due to heart failure) [9]. On the other hand, evidence of positive correlation was detected in other studies, including recent meta-analyses of 22 studies involving 22,428 non-diabetic patients; high HbA1c levels were associated with a higher rate of long-term death (OR = 1.76, 95% CI = 1.44–2.16) and myocardial infarction (OR = 1.69, 95% CI = 1.07–2.67). The findings for death remained the same after sensitivity analyses [10].

Moreover, HbA1c cut-off values for atherosclerosis, CAD diagnosis, and stages of coronary artery stenosis (CAS) show diverse results in the literature with evidence for increased CAD risk concomitant with increased HbA1c levels even in non-diabetic populations [11].

There is no available data regarding the above HbA1c cut-off values in the Saudi population. Our research for local HbA1c cut-off values for CAD diagnosis and its stages of severity can be of great help in optimizing the prevention of CAD and its sequelae, especially in high-CAD-risk groups like the Saudi population.

Our main aim is to investigate the role of the biomarker HbA1c as a predictor of CAD in non-diabetic patients with no previous established CAD diagnosis.

## Methods

### Study design and participants

This cross-sectional study included 38 patients who came to the outpatient clinic of Prince Sultan Cardiac Center, Al Qassim for evaluation of their chest pain. They were enrolled in the study between December 2017 and July 2018 after signing the informed consent that explained their rights in the study, the perceived risks and benefits of participation, and the measures taken by the investigators to keep their personal information confidential. The inclusion criterion was non-diabetic adults that had indication for CCTA. A patient was excluded from the study if he/she had any of the following: (a) previous CAD diagnosis, (b) impaired kidney function detected by urea and creatinine, (c) any end organ failure or malignancy, (d) active infection, (e) diabetes (HbA1c ≥6.5% or fasting glucose ≥126 mg/dL), (f) previous diabetes diagnosis, or (g) was using anti-diabetic medication and/or statins.

### Data collection and laboratory testing

Upon enrollment, the patients were interviewed with a standard questionnaire for the following data: (a) age (in years), (b) gender (male, female), (c) other demography, and (d) CAD risk factors, including diabetes, hypertension, dyslipidemia, physical inactivity, previous CAD, family history, and drug/substance intake. A basic physical examination was carried out, and an average of three resting blood pressure measurements were taken by a specialized cardiology nurse using a mercury sphygmomanometer. Hypertension was defined as blood pressure ≥ 140/90 mmHg or receiving anti-hypertension treatment. Body mass index (BMI) was calculated by dividing weight (kg) by height (meters squared). All patients had blood samples taken before CCTA for laboratory analysis, including urea, creatinine, fasting blood glucose (FBG), lipid profile, and HbA1c. HbA1c (Tina-quant Hemoglobin A1c Gen.3 REF 05336163 190 /Roche Diagnostics GmbH-Germany) was estimated according to the National Glycohemoglobin Standardization Program (NGSP) parameters. Criteria of diabetes mellitus diagnosis was defined according to the American Diabetic Association’s diagnostic criteria [12]: pre-diabetic stage [HbA1c 5.7–6.4 / impaired fasting glucose (IFG) (100–125 mg/dL)]; diabetes mellitus (HbA1c ≥6.5 /fasting glucose ≥126 mg/dL).

### CCTA

All patients had an appointment for evaluation of suspected ischemia via high resolution CCTA with a dual-source 256 slice scanner (Siemens Flash Definition CT scanner; Siemens, Berlin, Germany). After a calcium score scan, we used either a prospective electrocardiography (ECG) triggering or a retrospective ECG gating acquisition. Post-processing and reconstruction of the CCTA were carried out with a Multimodality Workplace (Siemens Medical Solutions, Erlangen, Germany).

### CCTA results interpretation

CCTA images were interpreted by two cardiologists who had at least 6 years of experience in CCTA interpretation. Based on the plaques detected in CCTA, CAS was quantified for each patient and was expressed in percentages [13]. Additionally, patients were classified as having minimal (< 25%), mild (25–49%), moderate (50–69%), and severe (≥70%) stenosis according to the degree of luminal obstruction [14].

### Statistical analysis

Quantitative variables are presented as mean ± SD. Qualitative variables are expressed as numbers and proportion. Correlation of HbA1c levels with CAS percentages and the number of affected vessels was determined by means of Spearman’s correlation test. A receptive operative characteristics (ROC) curve was generated to assess HbA1c between patients with and without coronary atherosclerosis. A Box and Whisker’s plot was made to assess the HbA1c values with different levels of CAD stenosis. *P*-values less than 0.05 were considered statistically significant. Statistical analyses were performed using SPSS Statistics, version 25 (SPSS Inc., Chicago, Illinois, USA).

## Results

The sample’s mean age was 50.8 years (standard deviation, SD = 9.5), and mean BMI was 28.3 kg/m^2^ (SD =5.8). Sixty-three percent (63%) were male, 65.5% were overweight or obese, 29.4% were hypertensive, and 21.4% had an above normal cholesterol level (> 200 mg/dl).

The HbA1c level was 5.7 ± 0.45% (4.7–6.4%), and the data indicated that 44.7% were normal (< 5.6%) and 55.3% were pre-diabetic (5.7–6.4%) (Table 1).

**Table 1:** Patient characteristics of the sample (*n* = 38)

Variable	Mean ± SD/ N (%)
Age (years)	50.87 ± 9.56
Male	24 (63.2)
Overweight/Obese	19 (65.5)
Hypertensive	5 (29.4)
High cholesterol	6 (21.4)
HbA1c (%)	
Normal	17 (44.7)
Pre-diabetic	21 (55.3)

Eighteen patients showed no CAS (47.4%), 12 showed minimal stenosis (31.6%), 3 showed mild stenosis (7.9%), 3 showed moderate stenosis (7.9%), and 2 showed severe stenosis (5.3%). The mean number of vessels affected was 0.84 (SD 0.95; range 0–3) (Table 2).

**Table 2:** Distribution of CAS among patients (*n* = 38)

Variable	Mean ± SD/ N (%)	Min-Max
Stenosis (%)	19.6 ± 19	5–75
Number of affected vessels	0.84 ± 0.95	0–3
Stenosis severity		
Normal	18 (47.37)	
Minimal	12 (31.58)	
Mild	3 (7.89)	
Moderate	3 (7.89)	
Severe	2 (5.27)	

**Fig. 1:**
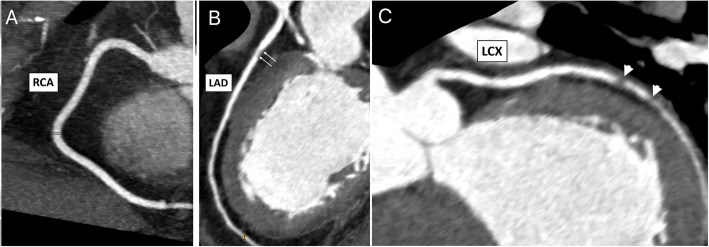
Mutiplaner reconstruction CCTA images for 3 studied cases with rising HbA1c levels. **a**. Normal right coronary artery (RCA), HbA1c = 4.8%; **b** Mild stenosis at proximal left descending coronary artery LAD stenosis, HbA1c = 5.8%; and (**c**) Severe stenosis of the left circumflex coronary artery (LCX), HbA1c = 6.3%

Figure 1 shows three examples of CCTA images with their related HbA1c levels.

HbA1c was not significantly correlated with CVD risk factors like age, systemic hypertension, or the serum cholesterol level, and it had only a mild correlation with BMI (0.4, *P* < 0.05).

A moderate correlation could be detected between HbA1c and CAS quantitative percentage (*r* = 0.470, *p* < =0.051) as well as between HbA1c and the number of affected coronary vessels (*r* = 0.5344, *p* < =0.0011) (Table 3).

**Table 3:** Correlation of HbA1c with CAS percentage & number of affected vessels

	Percentage of stenosis	Number of vessels
Spearman’s*r*-value	0.47	0.53
*p*-value	< 0.05	< 0.001
N	38	38

**Fig. 2:**
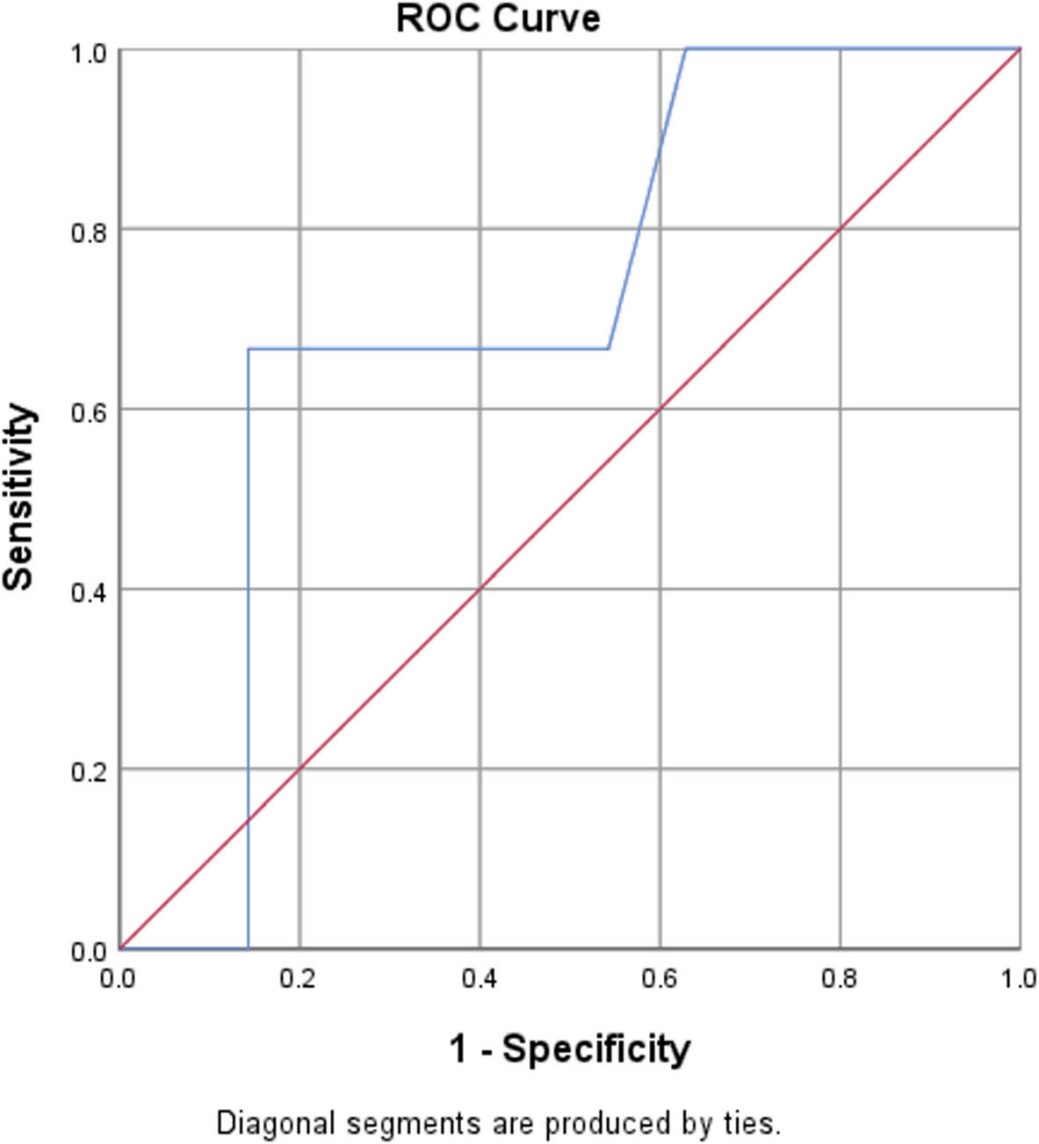
ROC curve for prediction of CAS using glycated hemoglobin level

A receiver operator characteristic (ROC) curve was generated to detect the ability of the HbA1c biomarker to be used as an early predictor for CAS in non-diabetics. The area under the curve was 71% (95% CI; 64–96%), with the best cut-off value of HbA1c at 5.9% (sensitivity 67% & specificity 74%) (Fig. 2).

## Discussion

A salient finding of this study was a moderate correlation between HbA1c and CAS percentages among non-diabetic patients. This finding is in agreement with a recent study by Ikeda et al. [15] that showed a higher HbA1c level as an independent risk factor of CAS. Previous studies, some of which included meta-analysis, found a positive correlation between HbA1c and cardiovascular events in non-diabetic adults [16–18]; however, these studies did not test the correlation between HbA1c and severity of CAS based on CCTA findings.

Geng et al. [10] conducted a meta-analysis using 20 studies involving 22,428 patients to investigate HbA1c correlation with clinical CAD outcomes; they found that an elevated HbA1c level increased the risks of both long-term mortality (odds ratio 1.76, 95% confidence interval 1.44–2.16, *p* < .001) and myocardial infarction (MI, odds ratio 1.69, 95% confidence interval 1.07–2.67, *p* = .026), but not the risk of early death in non-diabetic patients with CAD. More recently, Haring et al. [19] showed a positive association between high-normal HbA1c levels and increased CVD risk and mortality. On the other hand, there are studies that do not support that notion, such as by Liu et al. [20] and Shin et al. [21], in which HbA1c was not associated with prognosis among non-diabetic patients with myocardial infarction.

A number of factors may explain the discrepancy in results between these studies. Some studies recruited young people with favorable cardiac risk profiles, while other studies had more elderly participants with already advanced CAD complications [20]. A few studies resorted to retrospective design, and therefore, may have missed unrecorded adverse cardiac events [22]. The studies also varied in the outcomes that they were interested in, which ranged from very early changes in the arterial wall pathology [15], to short-term clinical adverse outcomes [23],to long-term morbidity and mortality [10].

This study’s finding of an HbA1c cut-off value of 5.9% to differentiate coronary stenosis among non-diabetic patients is supported by findings from other studies although there is no Saudi data available for comparison. Ashraf et al. [24] studied 299 non-diabetic patients who had coronary angiography for suspected ischemia and reported that anHbA1c level of 5.6% could be used for CAD-specific risk stratification (OR: 2.8, 95% CI: 1.3–6.2, *p*-value: 0.009). Tomizawa et al. [25] found that an HbA1c level above 6% was associated with significant CVD risk in non-diabetic patients.

Our study focused on pre-diabetic patients, who are usually overlooked and neglected in clinical practice although endothelial dysfunction, the main pathogenesis of both micro and macrovascular diabetic complications, starts to develop in this early stage [26]. Consequently, this study highlights the significance of sub-threshold levels of HbA1c in a pre-diabetic condition as an early predictor of CAS.

A recently published study by Engel et al. [26] compared the endothelial permeability, which is considered a hallmark of CAD, with different HbA1c levels using an albumin-binding MR probe. This cross-sectional study included 26 patients and concluded that patients with both intermediate and high HbA1clevels are associated with a larger extent of endothelial damage of the coronary arteries as compared to patients with HbA1c levels below 5.7% [27].

Abnormal glucose regulation (AGR) and insulin resistance are thought to be crucial factors for the development of subclinical atherosclerosis and, consequently, CAD. In fact, AGR has been independently associated with acute coronary events [23]. Additionally, insulin resistance and CAD have been found to co-exist in newly diagnosed patients with impaired glucose tolerance and impaired fasting glucose [28].

The increase in serum HbA1c is likely to be associated with the increase in the number of coronary vessels affected with stenosis. As the study by Haring et al. [19] found a positive link between high-normal HbA1c levels and increased risk of subclinical atherosclerosis (0.02 mm increase in the thickness of common carotid artery media per 1% increase in HbA1c), so did we find a positive correlation between serum HbA1c and the affected number of coronary vessels with stenosis. Our results further corroborated the findings of the study by Tomizawa et al. [25],who investigated the relationship of HbA1c and coronary plaque characteristics and reported that plaque formation in the coronary vessels was twice as likely (OR 2.19, 95% CI =1.37–3.45, *p* = 0.005) in patients with elevated HbA1c levels.

This study was the first in Saudi Arabia to assess the relationship between serum HbA1c and CAS by CCTA. Moreover, its participants were non-diabetic with relatively low CAD risk, so the study showed the role HbA1c could play in the prevention and monitoring of CAD among those who are otherwise free from cardiac events. Finally, HbA1c, which was the focus of this study, is a standardized and widely available test that can be easily employed in primary healthcare centers for early prevention of CAD events.

### Limitations

This study has few limitations. It was a single-center study and therefore may not have enrolled a full spectrum of pre-diabetic patients. It also had a small sample size. Future studies should employ larger samples and enroll patients from multiple sites to validate the findings of this study. Finally, the cross-sectional nature of this study with no follow up of the patients precluded it from establishing a temporal relationship between HbA1c and CAD. Future studies should employ a cohort design to follow up the patients. Additionally, trials could be undertaken to test the effect of lowering HbA1c on CAS in pre-diabetic patients.

## Conclusions

Based on our findings, we conclude that glycated hemoglobin A1c can be used as a predictive biomarker for CAD in non-diabetic patients with a cut-off value of 5.9%.

## Data Availability

The datasets used and/or analyzed during the current study are available from the corresponding author on reasonable request.

## Abbreviations

AGR: Abnormal glucose regulation
BMI: Body mass index
CAD: Coronary artery disease
CAS: Coronary artery stenosis
CCTA: Coronary computed tomography angiography
CVD: Cardiovascular disease
ECG: Electrocardiography
FBG: Fasting blood glucose
HbA1c: Glycated hemoglobin
IFG: Impaired fasting glucose
LAD: Left anterior descending coronary artery
LCX: Left circumflex coronary artery
MPR: Mutiplaner reconstruction
NFG: Normal fasting glucose
NGSP: National Glycohemoglobin Standardization Program
RCA: Right coronary artery
ROC: Receiver operator characteristic curve
